# Investigating the Effect of Intermittent Catheter Lubricant Type on the Transfer of Urinary Tract-Related Bacteria Using an In Vitro Bacterial Displacement Test

**DOI:** 10.7759/cureus.97743

**Published:** 2025-11-25

**Authors:** Kate Meredith, Victoria Mason, David Pollard, Ased Ali

**Affiliations:** 1 Research and Development, Convatec, Deeside, GBR; 2 Medical Affairs, Convatec, Deeside, GBR

**Keywords:** bacterial displacement, intermittent catheter, urinary catheter, urinary catheterisation, urinary tract infection

## Abstract

Aims

Whilst intermittent catheters (ICs) are the gold standard for managing bladder voiding difficulties, they are still linked with catheter-associated urinary tract infections (CAUTI). There are two main types of catheters: uncoated catheters, which require lubrication with a gel, and hydrophilic catheters, which are lubricated with water-based wetting solutions. Using a previously validated bacterial displacement *in vitro *model, we evaluated whether gel-based catheter lubricating products reduce bacterial displacement compared to water-based catheter lubricating products.

Methods and results

Simulated urethra agar channels (UACs) were prepared with catheter-specific-sized channels in selective media specific to the challenge organisms. Before insertion of ICs into the UACs, *Escherichia coli* and *Enterococcus faecalis* were separately inoculated in the first 1 cm of the UACs to represent the naturally contaminated distal urethra (meatus). Following insertion of the test catheter, UACs were processed for total viable counts (TVCs) or visual displacement images. Four ICs were evaluated: two gel-based ICs, Cure Twist^®^ (Gel-based female (GBF); Cure Medical, Henderson, NV, USA) and Cure Ultra^® ^(Gel-based male (GBM); Cure Medical, Henderson, NV, USA), and two water-based ICs, SpeediCath^®^ Compact Eve (Water-based female (WBF); Coloplast Ltd., Orton, Peterborough, UK) and SpeediCath^®^ Flex (Water-based male (WBM); Coloplast Ltd., Orton, Peterborough, UK). Both the microscopy results and TVCs showed that ICs lubricated with gel carried fewer bacteria along the length of the UAC and catheter in comparison to ICs lubricated with water.

Conclusions

The previously validated bacterial displacement method demonstrated that ICs with gel-based lubricants displaced fewer bacteria from the distal urethra to the proximal bladder, compared to ICs with water-based lubricants.

## Introduction

Catheter-associated urinary tract infections (CAUTIs) are associated with a significant burden on healthcare systems, impacting patient outcomes, healthcare costs and overall quality of life [1, 2]. They are a leading cause of healthcare-associated infections across the globe [2, 3], accounting for an estimated 449,334 urinary tract infections (UTIs) per year in the United States (U.S.) alone [4]. It is estimated that in the U.S., a single CAUTI infection alone could cost over $1000 to treat, and with the high numbers observed in the U.S., this is an extremely high financial burden on healthcare systems [5, 6]. The risk and nature of CAUTI differ depending on whether indwelling catheters or intermittent catheters (ICs) are used. The extended dwell time, commonly greater than 28 days, associated with indwelling catheters can lead to bacterial colonisation of the surface of the catheter and formation of biofilms [7]. The most troublesome of these is crystalline biofilms, produced by urease-producing bacteria, such as *Proteus mirabilis* [8]. Encrustation by these biofilms can lead to occlusion of the catheter lumen, which blocks urine flow and ultimately results in serious clinical complications. In contrast, ICs are designed for single use and are used several times over the course of the day (four to six times), as required by the user [9]. Intermittent catheterisation is widely considered the gold standard method for individuals with bladder voiding difficulties due to obstruction or dysfunction [10, 11] and allows users to have more freedom and greater autonomy [12].

Despite the advantages of ICs compared to indwelling catheters, ICs are still associated with significant levels of UTI. These complications result in significant healthcare resource consumption [13]. Intermittent catheterisation is a risk factor for UTI for several reasons. Firstly, it allows for the movement of the bacteria from the meatus, the entrance of the urethra where bacteria naturally occur, to be deposited into the bladder, without any mechanical rinsing of the bladder that would occur during normal voiding [7, 14]. Secondly, improper hygiene techniques from the user can result in contamination from the hands, skin or environment [14, 15]. Thirdly, ICs can cause microtrauma to the urethra, which may allow bacteria to penetrate the tissue [14]. *Escherichia coli *is the most commonly associated bacterium with general UTIs [16] and CAUTI [9, 17]. Catheter-associated UTI pathogens tend to be more resistant to empirical antibiotics, making infections harder to treat than non-CAUTI UTIs [18]. In the D’Incau et al. (2023) study, many of the *Escherichia coli* (*E. coli) *isolates were shown to be resistant to various tested antibiotics, including third-generation cephalosporins [18]. With this in mind, there is a critical need for antibiotic stewardship to drive the development of IC designs that will reduce the incidence of CAUTIs and minimise the need for antibiotics.

There are two main types of IC: uncoated (hydrophobic), which are lubricated with a gel and coated (hydrophilic) catheters lubricated with water-based wetting solutions. Introducing infection prevention features into the catheter product design may reduce the bacterial displacement of uropathogens from the distal urethra to the proximal end of the urinary tract, thus minimising the risk of infection. New innovations, such as coating-free urinary catheters that possess an integrated amphiphilic surfactant, have been shown to reduce urethral microtrauma compared to hydrophilic coated catheters, which could lead to a lower risk of infection [19]. Catheter insertion tips with a closed design have been shown to provide an effective bacterial barrier by reducing the level of colonisation at the proximal end of the urethra to non-detectable levels in a simulated urinary tract test model [20]. This compares favourably to insertion aids with an open design, which have shown limited protection against bacterial displacement during catheter insertion. The same study also identified that the majority of the bacterial cells were found in the gel lubricant, suggesting that the gel may play an important role in controlling bacterial transfer during catheterisation. The first objective of this work was to use the bacterial displacement *in vitro* test model, utilised by Meredith et al. (2024), to evaluate whether catheters lubricated with gels can reduce bacterial displacement compared to catheter products lubricated with water [20]. The second objective was to assess whether bacteria are able to move across catheter lubricating gels.

Some of the results captured in this article were previously presented as a meeting abstract at the 2024 International Continence Society (ICS) Conference from 23-25 October 2024.

## Materials and methods

Bacterial displacement testing

The bacterial displacement test method is a previously validated test method detailed in Meredith et al. (2024) [20].

Urethra Agar Channel Preparation and Challenge Organisms

For the bacterial displacement testing, *Escherichia coli* (*E. coli)* (National Collection of Industrial, Food and Marine Bacteria (NCIMB) 14067; NCIMB Ltd., Aberdeen, UK) (Gram-negative) and *E. faecalis* (National Collection of Type Cultures (NCTC) 12201; NCTC Ltd., Sailsbury, UK) (Gram-positive) were selected. To prepare an inoculum equivalent to ~1 x 10^8^ colony-forming units (CFU)/ml at an optical density of 540 nm, representative colonies were taken from an 18-24 h culture plate and dispersed in Maximum Recovery Diluent (MRD; Neogen Corporation, Lansing, MI, USA). A 1 x 10^7^ CFU/ml inoculum concentration was required for testing; therefore, the challenge inoculum was further diluted in MRD. 

Selective agars were used to aid visualisation of the bacteria: Harlequin Tryptone Bile X-Glucuronide (TBX) agar (Neogen Corporation, Lansing, MI, USA) with 0.4% Agar Bacteriological (AB; Oxoid Ltd., Basingstoke, Hampshire, UK) and Harlequin Vancomycin-Resistant Enterococcus (VRE) chromogenic agar (Neogen Corporation, Lansing, MI, USA) with 0.8% AB were used to prepare simulated urethra agar channels (UACs) for *E. coli* and *E. faecalis*, respectively. On Harlequin TBX agar with 0.4% AB, *E. coli* colonies appear blue-green, and on Harlequin VRE chromogenic agar with 0.8% AB, *E. faecalis* colonies appear dark green. Thirty millilitres of the above selective agar were dispensed into sterile 30 ml universal containers whilst in their molten state, and channels were created down the centre of the agar by placing sterile stainless-steel rods (4 mm diameter), held in place centrally by stainless-steel locating discs, into the container and removing once set. The UACs were left, standing, with the container lids removed within a laminar flow cabinet to allow excess fluid in the channel to evaporate and to ensure the channel was dry. Any excess fluid was removed with a sterile needle and syringe and swab as per the Meredith et al. (2024) publication [20].

Test Catheters

Four catheters were evaluated: Gel-based CH12 catheters, Cure Twist^®^ (Gel-based female (GBF); Cure Medical, Henderson, NV, USA) and Cure Ultra^®^ (Gel-based male (GBM); Cure Medical, Henderson, NV, USA), and water-based CH12 catheters, SpeediCath^®^ Compact Eve (Water-based female (WBF); Coloplast Ltd., Orton, Peterborough, UK) and SpeediCath^®^ Flex (Water-based male (WBM); Coloplast Ltd., Orton, Peterborough, UK).

Inoculation of UACs and Catheter Insertion

**Figure 1:**
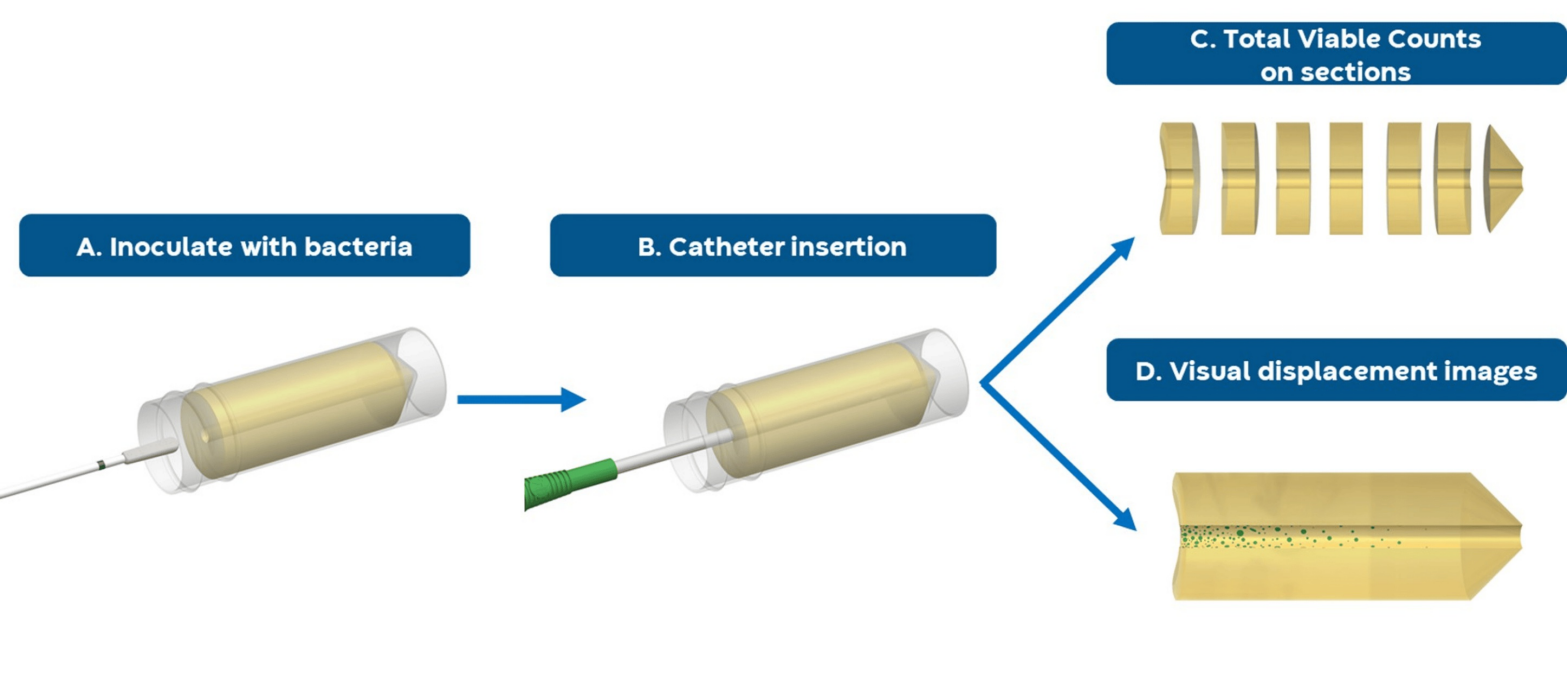
Bacterial displacement method schematic Bacteria were inoculated into the first 1 cm of the UAC, to represent the meatus (A). The catheter was then inserted and remained in place for 2 min, approximate urination time (B). The UACs were processed by cutting into sections and performing TVCs (C) or allowed to grow at body temperature for 24 h and visualised (D). TVC, total viable counts; UAC, urethra agar channel.

Fifty-microlitre aliquots of the inoculum were dispensed into individual sterile bijou containers, and a sterile flocked swab was dipped into the container for 10 s to absorb the inoculum. The swab containing the inoculum was inserted 1 cm into the insertion entry of a UAC and rotated in an anticlockwise direction twice before removing (Figure 1A). The test sample was removed, as per instructions for use (IFU), from the external packaging and inserted into the UAC. After leaving the sample in place within the UAC for 2 min (to represent the approximate urination time; Figure 1B), the sample was removed from the UAC and discarded. A bacterial growth control (with no catheter insertion) was prepared to ensure no alternate factors caused bacterial movement along the UAC and to ensure the channels were appropriately prepared. 

Urethral Agar Channel Processing: Total Viable Counts and Visual Displacement Images

Following inoculation of UACs and insertion of the catheter, the UACs were processed in one of two ways: for total viable counts (TVCs) (Figure 1C) or for visual displacement images (Figure 1D). For each of the two routes, five replicates for each of the samples for each of the challenge organisms were prepared.

For the processing of the TVCs, following inoculation and catheter insertion, using aseptic technique, the UACs were removed from their container and cut into 7 x 1 cm sections. The second, fourth and sixth centimetres (from the channel's entrance) were discarded while the remaining sections were individually homogenised in Dey-Engley Neutralising Broth (DENB; Neogen Corporation, Lansing, MI, USA) for 4 min on high within a laboratory blender to remove the bacteria from the surface of the agar and release them into the solution. Serial dilutions were carried out on the resulting suspension for each section separately, and appropriate dilutions were pipetted onto Tryptone Soy Agar (TSA; Neogen Corporation, Lansing, MI, USA) plates, which were then incubated for at least 48 h at 35 ± 3°C.

For visual displacement images, the channels were incubated for 24 h at 35 ± 3°C horizontally. After the 24 h incubation period, the UACs were removed from the incubator, the agar channel was removed from the container and split lengthwise directly down the centre and opened to allow images to be taken using a camera (Canon Model EOS450D, Tokyo, Japan).

Confocal Laser Scanning Microscopy

In a separate test, fluorescently tagged *E. coli* (ATCC-25922, LGC Group, Teddington, UK) were inoculated in the first 1 cm of the UAC, test catheters were inserted as per the above test, and then imaged with a confocal laser scanning microscope (LSM800 with Airyscan, Carl Zeiss, Germany). Eight images along the catheters were taken, observing the positioning of the *E. coli.* Imaging was performed at ×63 oil immersion magnification using the 488 nm laser and light transmitted microscopy to allow for positioning of the bacteria within the gel/water or surface of the catheter.

Statistical Analysis

Any statistically significant differences between each test catheter obtained during the bacterial displacement test were determined by a two-sample t-test, performed on Microsoft Excel (Microsoft Corp., Redmond, WA, USA).

Physical barrier testing

This test was performed as an adaptation of the bacterial displacement test. The channels were split in half lengthwise and filled with the lubricant type, i.e. sterile water or the gel (same as used in gel-based ICs). The aim was to establish if the bacteria could move across the lubricant-filled channels. 

Challenge organisms and UACs were prepared as stated above for the bacterial displacement testing. The UACs were aseptically removed from the container onto a sterile surface, and under aseptic conditions, the UACs were split in half lengthwise. The two halves of the UACs were then separated so the inside of the channel was visible. The visible channel was filled with 300 µl (enough to fill the channel without an overspill on the neighbouring agar) of the lubricant gel or sterile deionised water (SDW) and placed into a sterile petri dish. A sterile flocked swab was placed into the 50 µl inoculum of the above working concentration and left to sit for 10 s. The inoculated swab was then swiped along one of the inner edges of the UAC, along the side next to the channel, not within it. This was performed for all the test sample replicates. The petri dish was parafilmed (SIGMA, Sigma-Aldrich Corporation, St. Louis, MO, USA) and placed in the incubator at 35 ± 3°C for approximately 24 h. This step was repeated for each replicate and each challenge organism. A negative control was carried out for both selective media, consisting of 300 µl of lubricating gel or SDW without inoculation. Following the incubation period, images were taken to observe the bacteria’s motility through the UACs. Three replicates for each of the samples for each of the challenge organisms were prepared.

## Results

Bacterial displacement testing: visualisation of bacterial displacement

**Figure 2:**
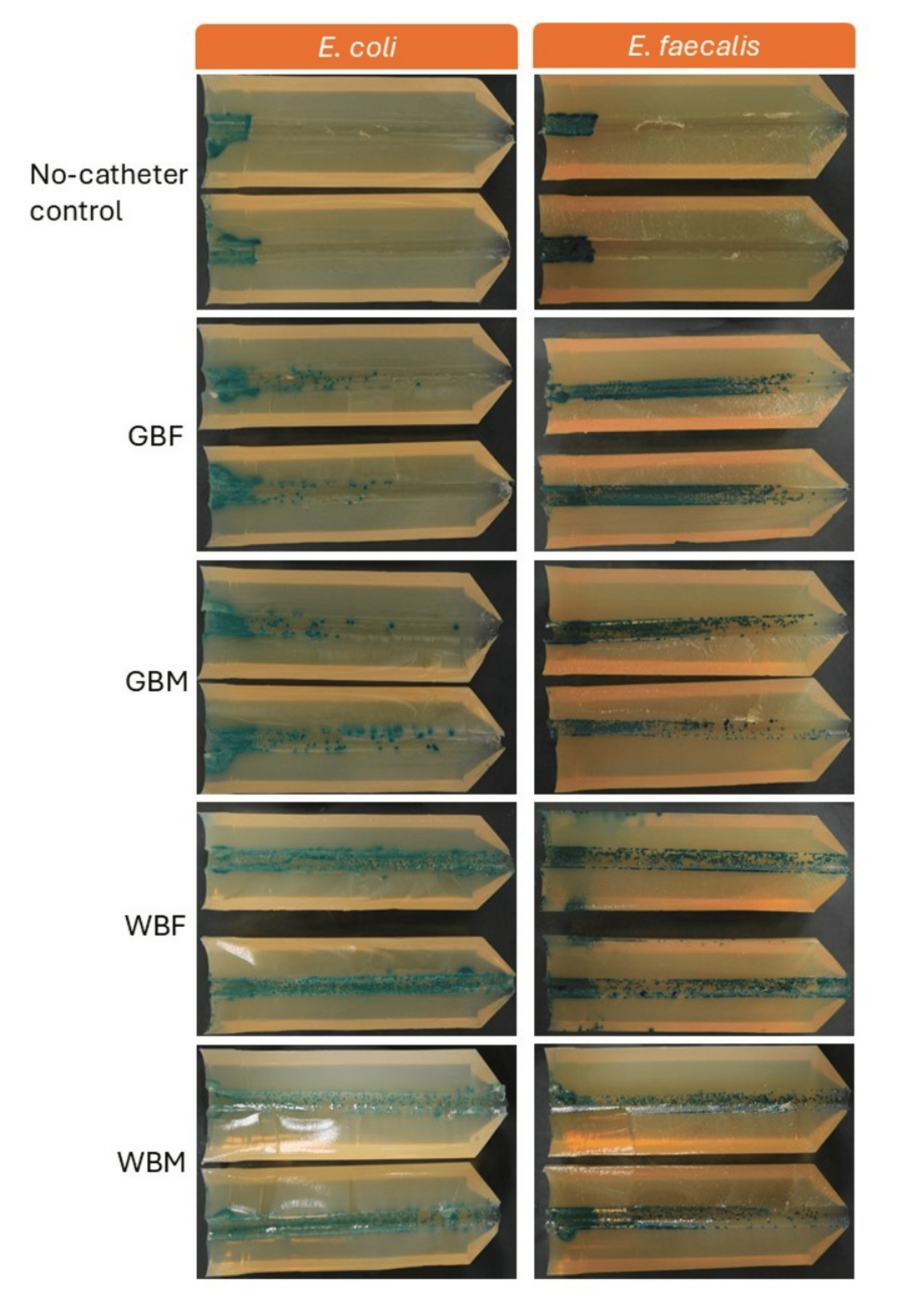
Representative visual displacement images (from five replicates) for E. coli and E. faecalis along UACs Each image shows both halves of the UAC following incubation. The left side of the UAC represents the insertion entry of the catheter, where applicable. *E. coli *was tested on Harlequin TBX agar (0.4% AB) showing blue-green colonies, and *E. faecalis* was tested on Harlequin VRE chromogenic agar (1% AB) showing dark green colonies. AB, Agar Bacteriological; GBF, gel-based female; GBM, gel-based male; TBX, Tryptone Bile X-Glucuronide; VRE, Vancomycin-Resistant Enterococcus; WBF, water-based female; WBM, water-based male; UAC, urethra agar channel.

Representative visual displacement images (from five replicates) for both challenge organisms are shown in Figure 2, where the left side of the UAC represents the insertion entry of the catheter. The area with the most growth, therefore the highest number of bacteria, shown in all images, was observed in the first 1 cm of the UACs. For the no-catheter bacterial growth control, the bacteria remained isolated to the first 1 cm of the UAC, demonstrating the absence of movement of bacteria by alternate factors and effective UAC preparation.

**Figure 3:**
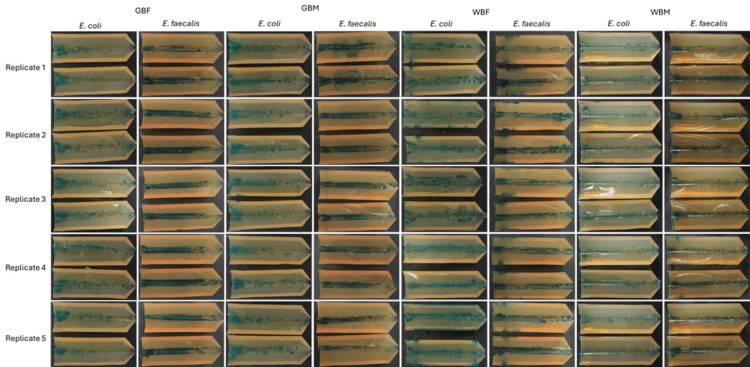
Visualisation of bacterial displacement for all five replicates for each catheter and challenge organism (E. coli and E. faecalis) along UACs Each image shows both halves of the UAC following incubation. The left side of the UAC represents the insertion entry of the catheter, where applicable. *E. coli *was tested on Harlequin TBX agar (0.4% AB) showing blue-green colonies, and *E. faecalis *was tested on Harlequin VRE chromogenic agar (1% AB), showing dark green colonies. AB, agar bacteriological; GBF, gel-based female; GBM, gel-based male; TBX, Tryptone Bile X-Glucuronide; VRE, Vancomycin-Resistant Enterococcus; WBF, water-based female; WBM, water-based male; UAC, urethra agar channel.

*E. coli* was displaced along the UAC to a lesser extent than *E. faecalis* for GBF and GBM test catheters. Both challenge organisms were displaced (moved) along the UACs to a similar extent for WBM and WBF test catheters. The WBM and WBF catheters displaced more of both challenge organisms along the UACs compared to the GBF and GBM test catheters, as observed by the presence of bacterial growth along the channel. Images of all five replicates for each catheter and challenge organism are shown in Figure 3.

Bacterial displacement testing: total viable counts

**Figure 4:**
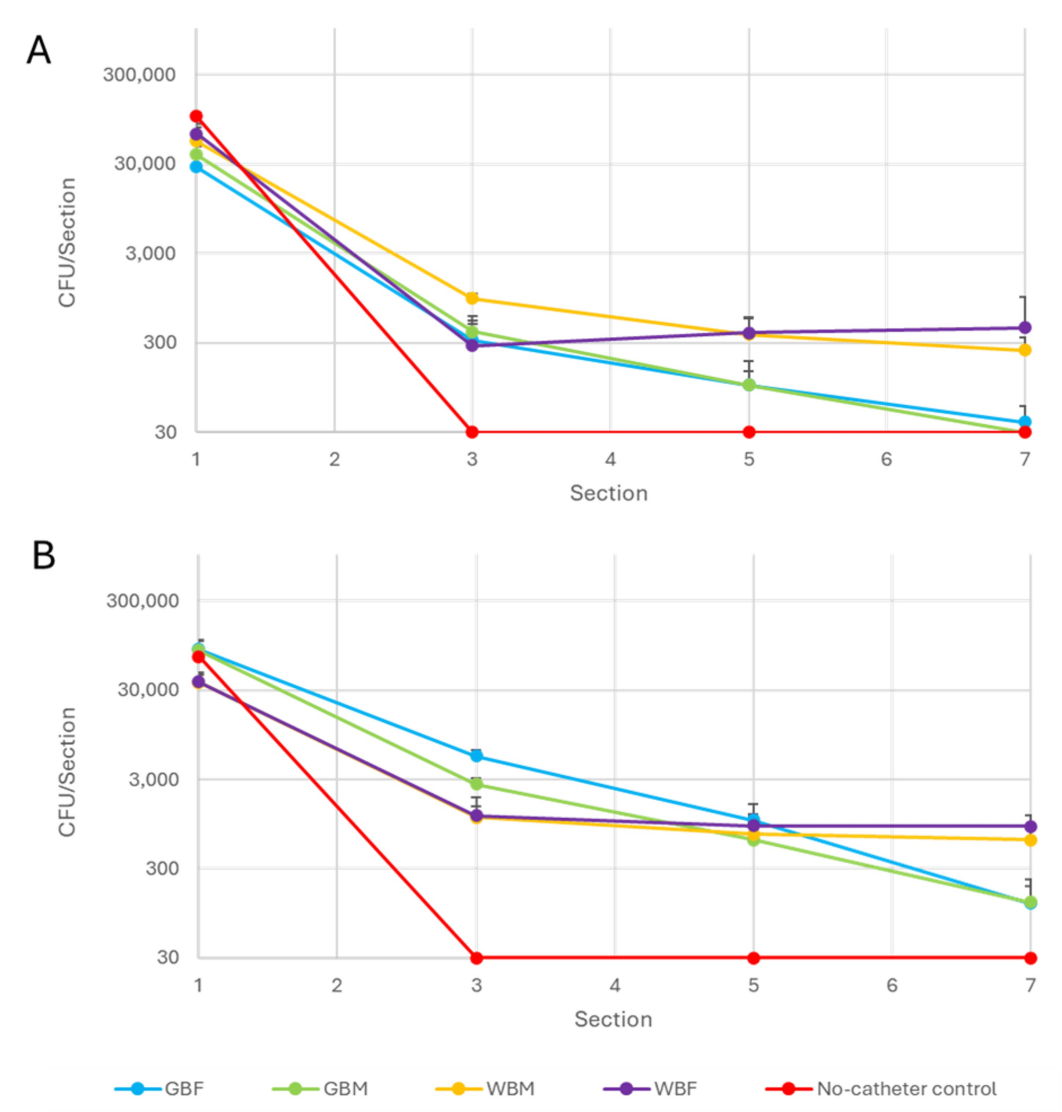
Bacterial displacement along the UACs for each tested IC *E. coli* (A) and *E. faecalis* (B) TVCs at sections 1, 3, 5 and 7 of the UAC. The detection limit of the test was 30 CFU. CFU, colony-forming units; IC, intermittent catheters; GBF, gel-based female; GBM, gel-based male; TVC, total viable counts; UAC, urethra agar channel; WBF, water-based female; WBM, water-based male.

TVCs (Figure 4) were comparable to the visual displacement images (Figure 2) of bacterial growth observed on the UACs. There was a downward trend in the bacterial presence along the UAC from section 1, where the highest number of bacteria was observed, to section 7, where the least bacteria was observed for both *E. coli* and *E. faecalis*. This difference between section 1 and section 7 was significant for all ICs tested for both challenge organisms (p<0.0001). Bacteria were detected in the first 1 cm of the no-catheter control, while undetectable (<30 CFU) levels of bacteria were observed along the remaining sections of the UAC, as expected, confirming UAC preparation was optimal.

There was a notable difference in the performance of gel-based ICs and water-based ICs in section 7 of the UACs. Both GBF and GBM ICs displaced fewer bacteria compared to WBM (p=0.0004 and p=0.0022) and WBF (p=0.0662 and p=0.0003) for *E. coli *and *E. faecalis*, respectively. Numbers of *E. coli* were undetectable for GBM and nearly undetectable for GBF (gel-based lubricated ICs) in section 7. These low numbers were not observed for the water-based lubricated ICs for* E. coli*. These small numbers were also not observed for *E. faecalis *for any of the ICs tested, where bacterial numbers were still present in section 7.

In section 1, there were also differences observed between the ICs. There were significantly more *E. coli *(p=0.00215) and *E. faecalis* (p=0.00035) bacteria in this section for GBF compared to WBF. This was also observed between the GBM and WBM ICs for *E. faecalis *(p=0.00105).

Physical barrier testing 

**Figure 5:**
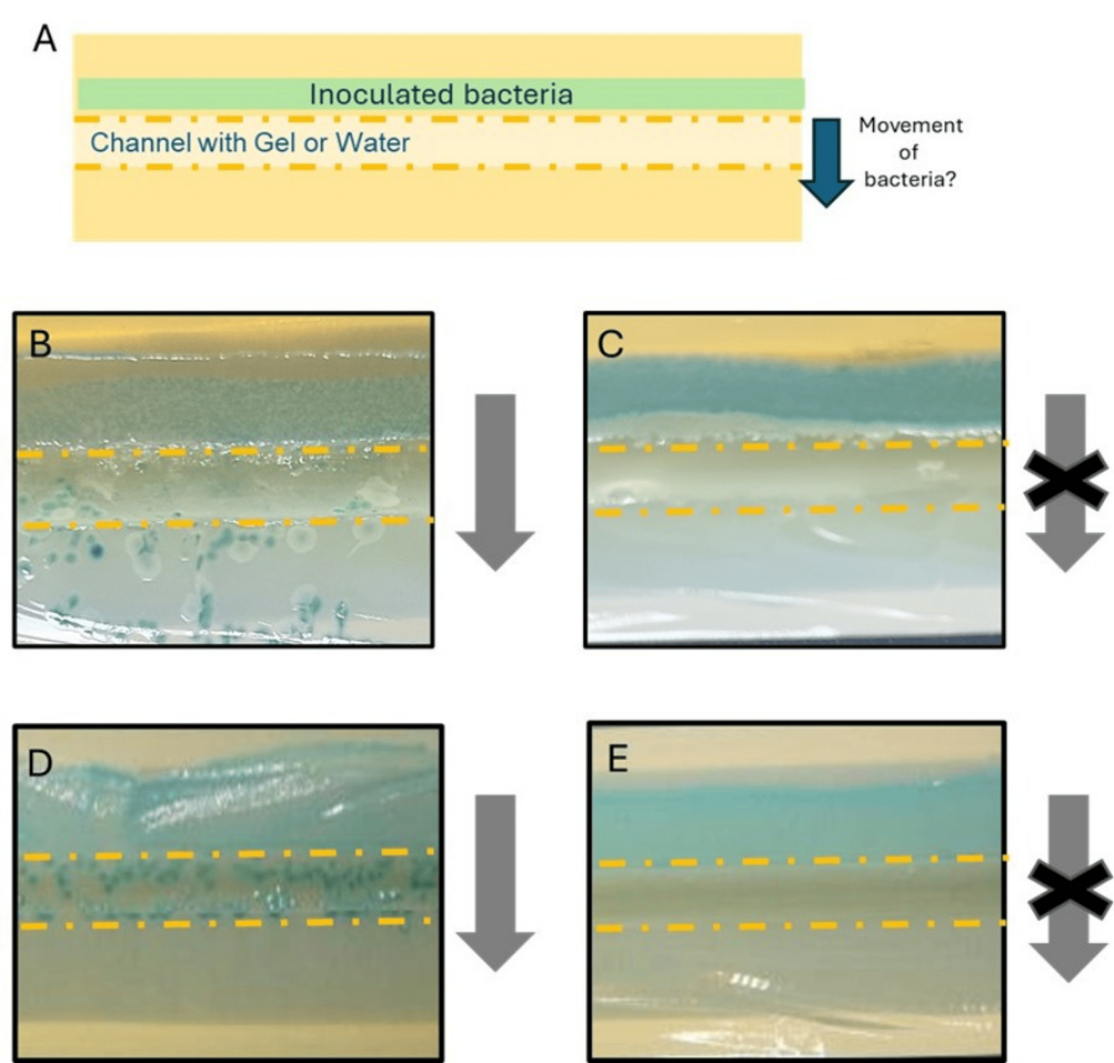
Representative images of the physical barrier testing Test setup (A). These representative images were magnified to facilitate clearer observation of bacterial growth. *E. coli *growth across the channel when filled with water (B) or gel (C). *E. faecalis* growth across the channel when filled with water (D) or gel (E). Orange dash line represents the edge of the channel, and lubrication samples were added within the channel. Grey arrow indicated whether bacteria were able to cross the water/gel sample.

To further understand the results observed within the bacterial displacement method, a supplementary test was developed using the UAC, but in contrast to the previous test, the channel was filled with gel or sterile water, and bacteria were inoculated along one side of the length of the channel (Figure 5A). Following incubation, bacterial growth was observed to establish if bacteria had moved across the lubricant-filled channels. For the water-filled channel, bacteria were present within the channel where the water was present and on the other side of the channel for both *E. coli *(Figure 5B) and *E. faecalis*, though slightly less (Figure 5D). Channels filled with the gel did not show growth for either *E. coli *(Figure 5C) or *E. faecalis* (Figure 5E) in any areas except where inoculated, indicating that the bacteria had not moved across the gel or into the gel.

**Figure 6:**
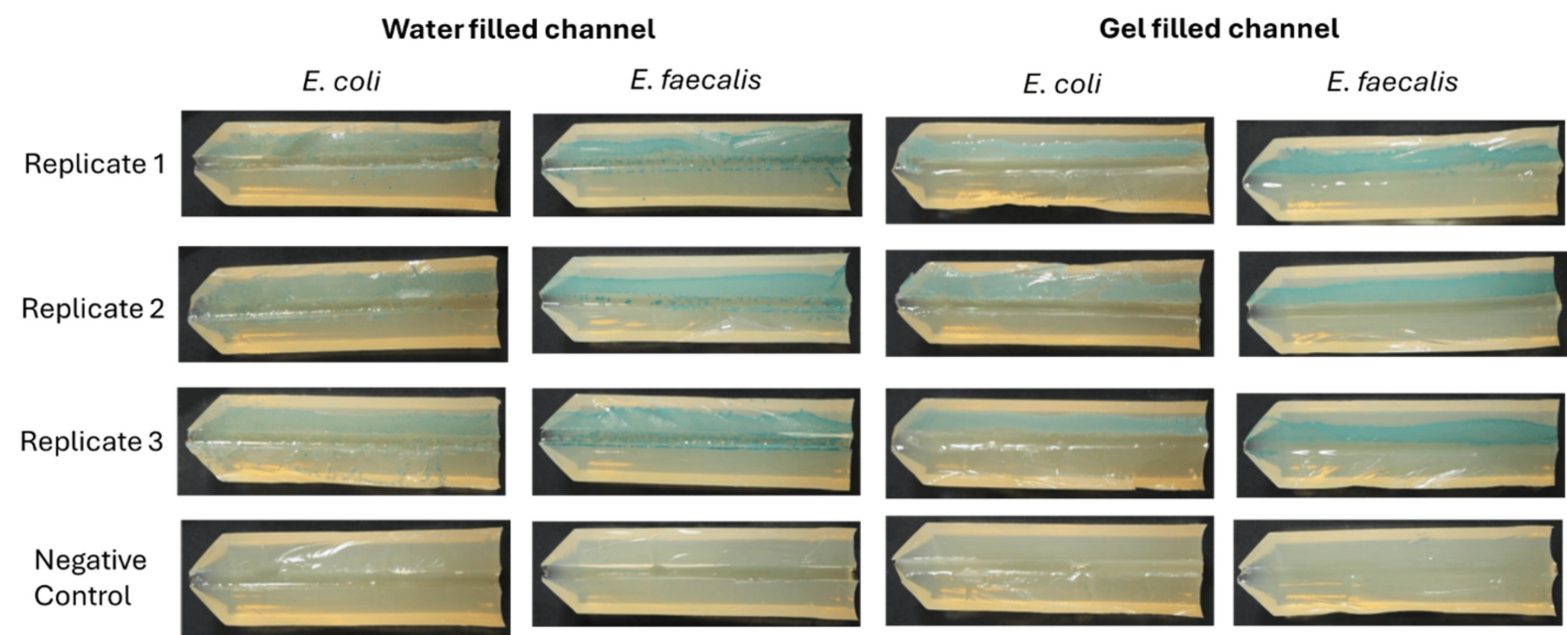
Full length UAC images of the physical barrier testing Images showing the full length of UAC and the presence or absence of growth. Three replicates were performed for *E. coli *and *E. faecalis *with channels filled with water or gel (as stated). UAC, uretha agar channel.

Images of all three replicates for each sample and challenge organism are shown in Figure 6.

Confocal laser scanning microscopy

**Figure 7:**
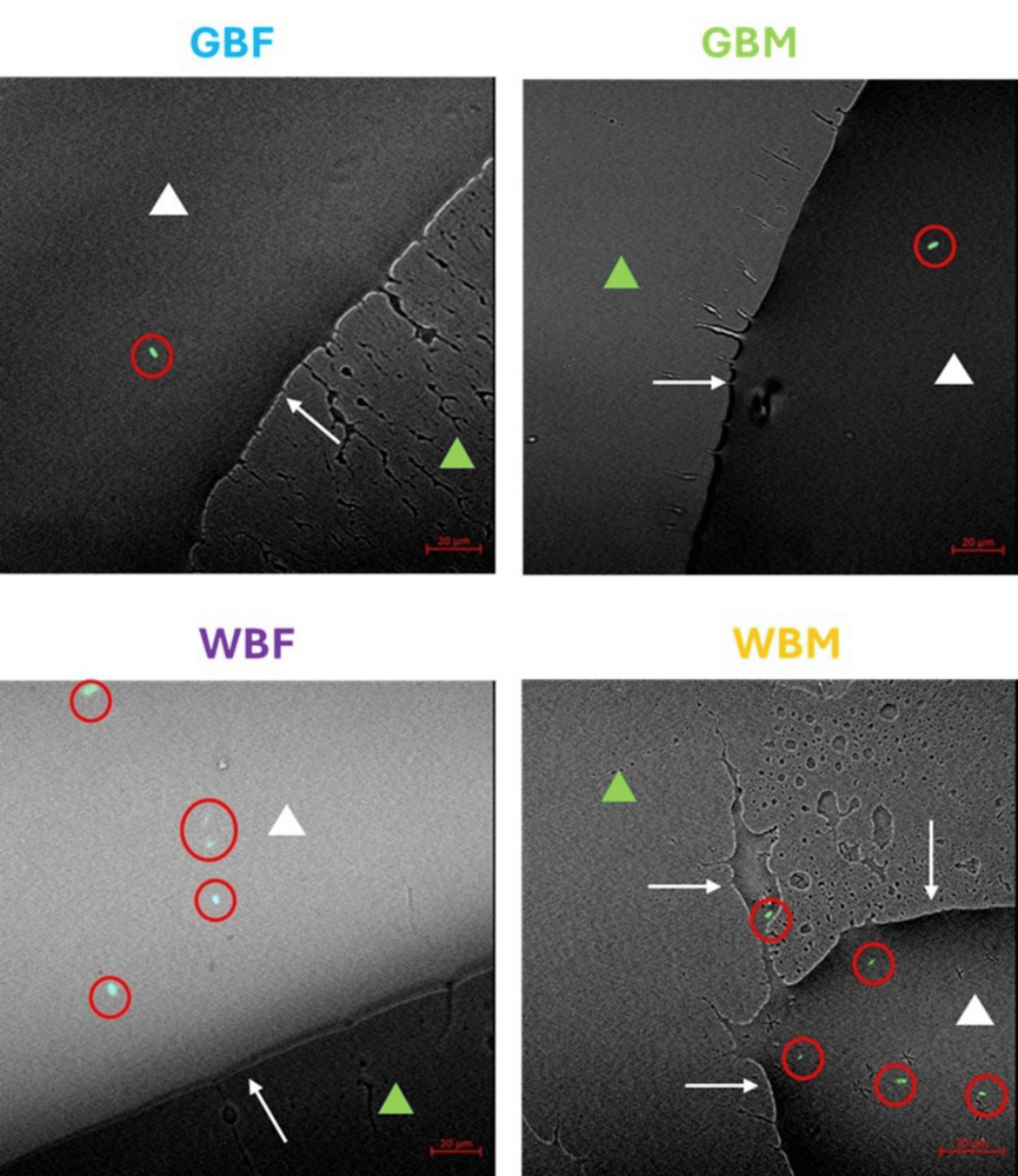
Representative confocal laser scanning microscopy images of the tested ICs Representative images showing the catheter surface (green triangle), lubricant used (gel or water; white triangle with white arrow highlighting the lubricant edge), and the presence of fluorescently tagged *E. coli *(green rods, red circle). GBF, gel-based female; GBM, gel-based male; IC, intermittent catheter; WBF, water-based female; WBM, water-based male.

The bacterial displacement test focuses on the number of bacteria displaced along the UAC. In contrast, here, using fluorescently tagged *E. coli*, the location and number of bacteria on the catheters were observed. Bacteria were observed on all the test catheters, with more observed in the water-based catheters compared to the gel-based catheters. Example images are shown in Figure 7. Bacteria were primarily found within the hydrating component rather than the surface of the catheter; however, it is worth noting that in the gel-based lubrication, the bacteria were primarily observed in larger areas of gel, while in the water-based lubricant, the bacteria were observed in both larger and smaller areas of lubricant.

**Figure 8:**
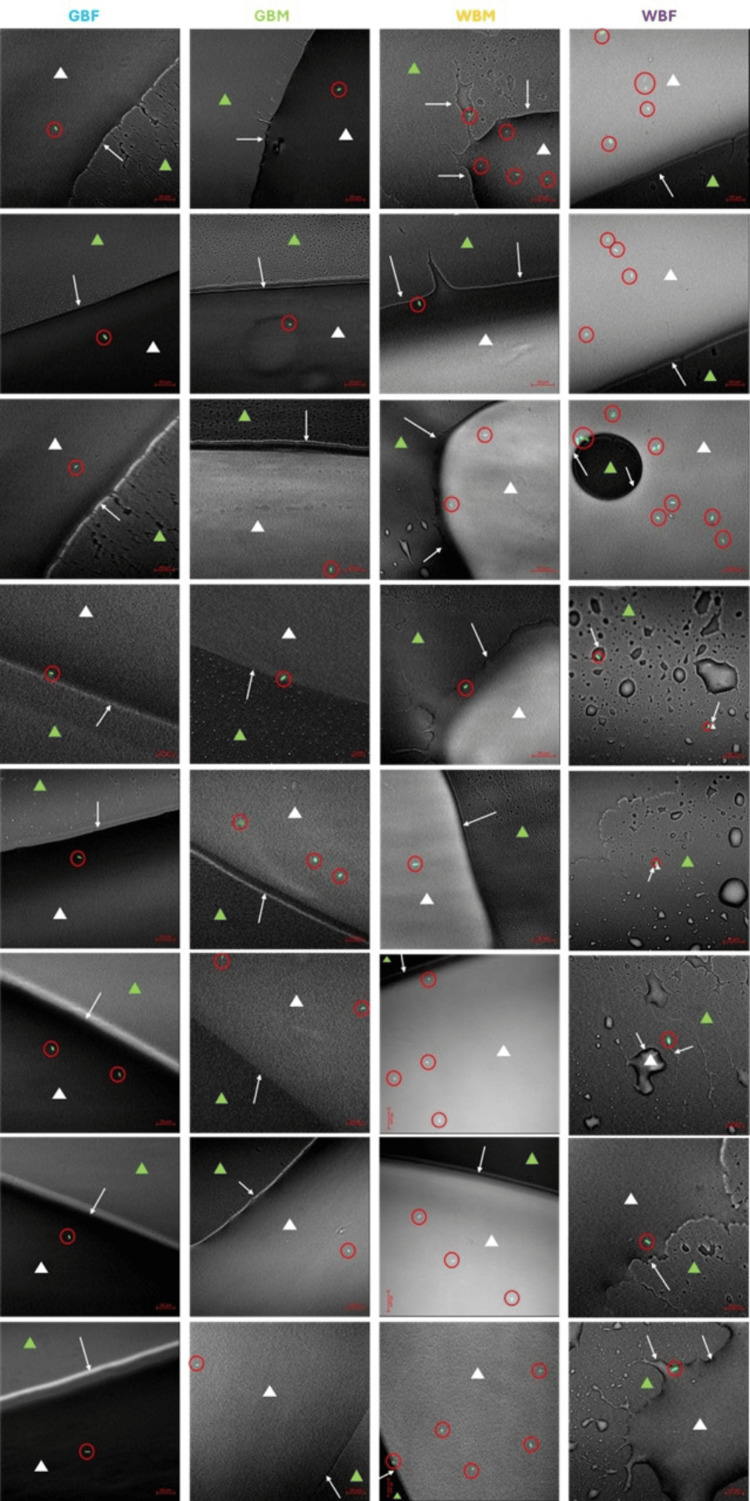
Confocal laser scanning microscopy images showing the eight replicates for each tested IC Images showing the catheter surface (green triangle), lubricant used (gel or water; white triangle with white arrow highlighting the lubricant edge), and the presence of fluorescently tagged *E. coli *(green rods, red circle). GBF, gel-based female; GBM, gel-based male; IC, intermittent catheter; WBF, water-based female; WBM, water-based male.

Images of all five replicates for each catheter and challenge organism are shown in Figure 8.

## Discussion

Using a previously developed *in vitro* bacterial displacement model [20], we assessed differences in IC lubricant type, water or gel, on bacterial displacement. We demonstrated, via microscopy and TVCs, that ICs lubricated with gel carry fewer bacteria along the length of the UAC in comparison to catheters lubricated with water. This observation could be clinically significant, as repeat insertions performed regularly by IC users increase the likelihood of bacteria moving from the meatus to the bladder, resulting in infection; therefore, reducing bacterial displacement through IC design may aid in reducing CAUTIs. These findings become increasingly important given the rising antimicrobial resistance among CAUTI pathogens. D'Incau et al. (2023) demonstrated widespread antibiotic resistance in CAUTI isolates, making prevention strategies critical [18]. By reducing bacterial translocation from the meatus to the bladder, gel-based lubrication could decrease both CAUTI incidence and selective pressure for resistant organisms, supporting antimicrobial stewardship goals.

Studies investigating the relationship between infection risk and localisation of bacterial load have established increased colonisation at the distal urethra, with the level of colonisation shown to progressively reduce as the distance of the meatus increases [21-23]. The primary reason for increased UTI reports in women compared to men is likely due to the shorter length of the female urethra, as a shorter urethra provides a shorter distance for the bacteria to travel and cause infection. Kunin et al. (2002) investigated the differences in bacterial colonisation for female subjects who do not suffer UTIs compared with female subjects who suffer from recurrent UTIs [23]. The bacterial count considerably decreased from the most distal section to the most proximal segment in women without UTIs compared to women with recurrent infections. These findings confirm that localisation of bacterial colonisation is a key contributor to normal UTI. As described by Kennelly et al. (2019), translocation of bacteria by the catheter tube during insertion and the absence of urethral rinsing during bladder voiding are key risk factors for IC users [14]. Combining the findings from Kennelly et al. (2019) and Kunin et al. (2002) [14, 23], the mean bacterial count along the urethra is likely to be less steep for IC users, boosting colonisation around the bladder neck and subsequently increasing the risk of infection.

It is difficult to compare ICs clinically as there are many factors that can cause CAUTIs, including poor insertion technique, poor hygienic procedure, gender, age and pre-existing health problems [14, 24]. There are limited *in vitro* microbiology tests which compare types of IC technologies, in connection with UTIs caused by bacteria [7, 20, 25] or physical features [19]. Only recently, a model developed by Dean et al. (2024) was published, which evaluated the bacterial transfer protective features of sleeve and insertion tips, and found that both aided in minimising bacteria transfer from the hands and the meatus to the catheter [25]. When considered alongside the insertion tip bacterial displacement study [20] and this publication evaluating gel vs water lubrication effects on bacteria movement from the meatus, it is clear that* in vitro* tests can provide information to aid future IC design.

Bacteria have different abilities to move on surfaces, aiding in infection. They can swim, swarm, twitch, slide, or glide depending on the structures and mechanisms found on their surface or within their genetics, e.g., flagella [26]. *E. coli* is motile and has many mechanisms to aid movement [27]. While *E. faecalis *is classed as non-motile and lacks common motility mechanisms, more recent research has shown that they are capable of some translocation and penetration of cell surfaces during infection [28]. It was apparent using the physical barrier test with gel or with water, that the motile *E. coli* bacteria were able to move more easily across the water channel compared to the gel channel. This movement was also seen with *E. faecalis,* but to a lesser extent compared to *E. coli*. We surmise that *E. faecalis* was taken by the water rather than moving itself. The gel was effective at reducing the movement of both motile and non-motile bacteria, likely through multiple mechanisms: physical entrapment in the polymer network, osmotic stress effects, and potential antimicrobial properties of gel preservatives. The confocal findings showing bacteria primarily in larger gel areas versus both large and small water areas support this entrapment hypothesis. The results suggest that the gel, likely due to its viscosity, prevented the bacteria from moving easily over the environment compared to water, acting like a barrier and potentially trapping the bacteria.

Limitations to this research should be noted. On a practical side, the bacterial displacement method, due to labour intensity, not all sections of the UAC were processed for TVCs. However, despite this limitation, there was a clear general downward trend in TVCs shown from the alternating UAC sections that were processed. From a more clinical point of view, this* in vitro* test does not consider urine flow dynamics, as no urine was passed through the catheter. During testing, there is only one insertion of the ICs per test, therefore representing a single-use scenario; this is not clinically representative, as users will potentially use catheters four to six times daily [9]. Additionally, the methods utilised are *in vitro *methods that cannot fully mimic a clinical situation and don’t account for other factors within the body that may aid the bacteria in causing an infection, such as catheter-induced inflammation, but also factors that might hinder the bacteria from causing an infection, e.g., host immune response.

The substantial reduction in bacterial displacement with gel-based catheters suggests potential for risk-stratified catheter selection. High-risk patients (neurogenic bladder, immunocompromised, recurrent UTI history) may benefit most from gel-based lubrication, while low-risk patients could reasonably use water-based catheters based on preference and cost considerations. Future guidelines could incorporate bacterial displacement potential into catheter selection algorithms.

This bacterial displacement test method has been used to evaluate IC insertion tips [20] and now the types of IC non-antimicrobial lubricants. Future testing could look to use the method to investigate further technologies, such as antimicrobial gels used for lubrication [29, 30] and their effect on bacterial displacement. Additionally, the testing could be expanded to include studying gels further and trying different viscosities that might have similar or more effective results. The spectrum of CAUTI-related bacteria could be expanded, including *Klebsiella spp.* and *Proteus spp.* [16]. As IC users perform repeat insertion during the day, this is another avenue that could be explored in the future.

## Conclusions

The *in vitro *UAC model was sensitive enough to evaluate different IC technologies and bacteria, aiding in the understanding of how different lubricants can affect bacterial movement. The study showed that ICs with gel-based lubricants displaced fewer bacteria from the distal urethra to the bladder compared to ICs with water-based lubricants, using a validated *in vitro *UAC model. Bacterial transfer from a contaminated meatus along the urethra can be reduced depending on the lubricant type and/or viscosity, with the increased viscosity of gel-based lubricants potentially trapping the bacteria to reduce their transfer.

## References

[1] Hearn JH, Selvarajah S, Kennedy P, Taylor J (2018). Stigma and self-management: an Interpretative Phenomenological Analysis of the impact of chronic recurrent urinary tract infections after spinal cord injury. Spinal Cord Ser Cases.

[2] Öztürk R, Murt A (2020). Epidemiology of urological infections: a global burden. World J Urol.

[3] King C, Garcia Alvarez L, Holmes A, Moore L, Galletly T, Aylin P (2012). Risk factors for healthcare-associated urinary tract infection and their applications in surveillance using hospital administrative data: a systematic review. J Hosp Infect.

[4] Gad MH, AbdelAziz HH (2021). Catheter-associated urinary tract infections in the adult patient group: a qualitative systematic review on the adopted preventative and interventional protocols from the literature. Cureus.

[5] Hutton DW, Krein SL, Saint S, Graves N, Kolli A, Lynem R, Mody L (2018). Economic evaluation of a catheter-associated urinary tract infection prevention program in nursing homes. J Am Geriatr Soc.

[6] McCleskey SG, Shek L, Grein J (2022). Economic evaluation of quality improvement interventions to prevent catheter-associated urinary tract infections in the hospital setting: a systematic review. BMJ Qual Saf.

[7] Barford JM, Anson K, Hu Y, Coates AR (2008). A model of catheter-associated urinary tract infection initiated by bacterial contamination of the catheter tip. BJU Int.

[8] Pelling H, Nzakizwanayo J, Milo S (2019). Bacterial biofilm formation on indwelling urethral catheters. Lett Appl Microbiol.

[9] Neumeier V, Stangl FP, Borer J (2023). Indwelling catheter vs intermittent catheterization: is there a difference in UTI susceptibility?. BMC Infect Dis.

[10] Yates A (2023). Intermittent self-catheterisation: the gold standard for individuals with bladder dysfunction. Br J Community Nurs.

[11] Ginsberg DA, Boone TB, Cameron AP (2021). The AUA/SUFU guideline on adult neurogenic lower urinary tract dysfunction: treatment and follow-up. J Urol.

[12] Blanc BF, Rodríguez-Almagro J, Lorenzo-García C (2021). Quality of life and autonomy in patients with intermittent bladder catheterization trained by specialized nurses. J Clin Med.

[13] Hasan SA, Neal-Herman L, Norman HS, Zhao JZ, Carlson A (2022). Patient support program and healthcare resource utilization in patients using clean intermittent catheterization for bladder management. J Wound Ostomy Continence Nurs.

[14] Kennelly M, Thiruchelvam N, Averbeck MA (2019). Adult neurogenic lower urinary tract dysfunction and intermittent catheterisation in a community setting: risk factors model for urinary tract infections. Adv Urol.

[15] Hudson E, Murahata RI (2005). The 'no-touch' method of intermittent urinary catheter insertion: can it reduce the risk of bacteria entering the bladder?. Spinal Cord.

[16] Flores-Mireles AL, Walker JN, Caparon M, Hultgren SJ (2015). Urinary tract infections: epidemiology, mechanisms of infection and treatment options. Nat Rev Microbiol.

[17] Dedeić-Ljubović A, Hukić M (2009). Catheter-related urinary tract infection in patients suffering from spinal cord injuries. Bosn J Basic Med Sci.

[18] D'Incau S, Atkinson A, Leitner L, Kronenberg A, Kessler TM, Marschall J (2023). Bacterial species and antimicrobial resistance differ between catheter and non-catheter-associated urinary tract infections: data from a national surveillance network. Antimicrob Steward Healthc Epidemiol.

[19] Burns J, Pollard D, Ali A, McCoy CP, Carson L, Wylie MP (2024). Comparing an integrated amphiphilic surfactant to traditional hydrophilic coatings for the reduction of catheter-associated urethral microtrauma. ACS Omega.

[20] Meredith K, Pollard D, Mason V, Ali A (2024). The bacterial displacement test: an in vitro microbiological test for the evaluation of intermittent catheters and urinary tract infection. J Appl Microbiol.

[21] Cox CE (1966). The urethra and its relationship to urinary tract infection: the flora of the normal female urethra. South Med J.

[22] Cox CE, Lacy SS, Hinman F Jr (1968). The urethra and its relationship to urinary tract infection. II. The urethral flora of the female with recurrent urinary infection. J Urol.

[23] Kunin CM, Evans C, Bartholomew D, Bates DG (2002). The antimicrobial defense mechanism of the female urethra: a reassessment. J Urol.

[24] Averbeck MA, Kennelly M, Thiruchelvam N (2023). Risk factors for urinary tract infections associated with lower quality of life among intermittent catheter users. Br J Nurs.

[25] Dean NL, Gras J, Lantz EE (2024). Microbial transfer by intermittent catheters: an in vitro evaluation of microbial transfer in catheter with variable protective features. J Wound Ostomy Continence Nurs.

[26] Zegadło K, Gieroń M, Żarnowiec P, Durlik-Popińska K, Kręcisz B, Kaca W, Czerwonka G (2023). Bacterial motility and its role in skin and wound infections. Int J Mol Sci.

[27] Zhou Y, Zhou Z, Zheng L (2023). Urinary tract infections caused by uropathogenic escherichia coli: mechanisms of infection and treatment options. Int J Mol Sci.

[28] Ramos Y, Sansone S, Hwang SM (2022). Remodeling of the enterococcal cell envelope during surface penetration promotes intrinsic resistance to stress. mBio.

[29] Cohen A (1985). A microbiological comparison of a povidone-iodine lubricating gel and a control as catheter lubricants. J Hosp Infect.

[30] Payne D, Kerrigan P (2020). One trust's rationale for choosing a lubrication gel for use in catheterisation. Br J Nurs.

